# Biocharts: a visual formalism for complex biological systems

**DOI:** 10.1098/rsif.2009.0457

**Published:** 2009-12-18

**Authors:** Hillel Kugler, Antti Larjo, David Harel

**Affiliations:** 1 Computational Biology Group, Microsoft Research, Cambridge, UK; 2 Department of Signal Processing, Tampere University of Technology, Finland; 3 Department of Computer Science and Applied Mathematics, The Weizmann Institute of Science, Rehovot, Israel

**Keywords:** biological modelling, multi-scale modelling, statecharts, bacterial chemotaxis, metabolism

## Abstract

We address one of the central issues in devising languages, methods and tools for the modelling and analysis of complex biological systems, that of linking high-level (e.g. intercellular) information with lower-level (e.g. intracellular) information. Adequate ways of dealing with this issue are crucial for understanding biological networks and pathways, which typically contain huge amounts of data that continue to grow as our knowledge and understanding of a system increases. Trying to comprehend such data using the standard methods currently in use is often virtually impossible. We propose a two-tier compound visual language, which we call *Biocharts*, that is geared towards building fully executable models of biological systems. One of the main goals of our approach is to enable biologists to actively participate in the computational modelling effort, in a natural way. The high-level part of our language is a version of statecharts, which have been shown to be extremely successful in software and systems engineering. The statecharts can be combined with any appropriately well-defined language (preferably a diagrammatic one) for specifying the low-level dynamics of the pathways and networks. We illustrate the language and our general modelling approach using the well-studied process of bacterial chemotaxis.

## 1. Introduction

Several notations have been introduced to formalize biological networks and metabolic pathways. Two of the best known of these are Kohn diagrams (Kohn & Aladjem 2006; Kohn *et al.* 2006) and Kitano process diagrams (Kitano *et al.* 2005), which were recently extended and unified as part of a community effort (Novère *et al.* 2009). These approaches attempt to go beyond the informal diagrams biologists typically use. They propose a syntax for the visual elements of the language, which is usually a graph with a variety of different flavours of arrows, and for some subset of the notation, they also provide an executable semantics. Some of these languages are supported by software tools, e.g. CellDesigner (Funahashi *et al.* 2003). By the term *executable semantics* for a visual language, we mean a precise way to produce a meaning for any given model, which is clear and rigorous and provides all that is required for executing the model on a computer and analysing the model's behaviour. This ability to execute biological models is a prerequisite to performing *in silico* experiments, gaining a system-level understanding of biological phenomena and making predictions that can be later validated experimentally.

The premise of the present paper is that when it comes to modelling and simulating large and complex pieces of biology, these approaches are less than perfect. First, remaining on the network and pathway level can be limiting when things involve more than a manageable number of excitatory or inhibitory arrows. One of the basic maxims of system and software engineering calls for modularity, abstraction and separation of concerns not only when dealing with the structure of a system but also for its behaviour: we need to be able to think, model and view the system's behaviour on various levels of detail.

Second, there is the technical issue of providing a natural executable semantics for very large diagrams (such as the diagram of the growth factor, EGFR's pathway in Oda *et al.* (2005) or the many incredibly large graphs of metabolic pathways that people have devised). Explaining the intuitive meaning of each icon in the language and mapping it to the relevant biological elements, as is done in Novère *et al.* (2009), is an important step in the definition of a visual language, but it falls short of an executable semantics. We claim that attempting to capture the full behaviour of a system by combinations of the dynamics of the single arrows is unnatural and non-intuitive. Sometimes, it can become impossible. How, for example, does one assert, naturally, and in a way that a viewer can comprehend easily, that some entire portion of the complex diagram starts ‘working’ only upon the occurrence of an event that is described in a totally different part of the diagram, and that some other event causes a subtle change in the way the said portion works? How does one depict (again, naturally) the difference between ‘chunks’ of behaviour that occur concurrently and those that may also occur independently? These and many other flavours of the reactive behaviour typical of complicated biological artefacts can benefit from having at hand a formalism that is expressively richer and more modular and comprehensible than pathway and network diagrams.

In this paper, we suggest a compound, two-tier visual language for constructing fully executable models of complex biological systems. Our language, *Biocharts*, is based on combining statecharts (Harel 1987), which capture the high-level state-based strata of system behaviour, with an appropriately well-defined language (preferably a diagrammatic one) for specifying the lower-level dynamics of the pathways and networks. One of the main goals of our approach is to enable biologists to actively participate in the computational modelling effort, in a natural way.

We illustrate Biocharts using the well-studied biological systems of bacterial chemotaxis (Wadhams & Armitage 2004) and metabolism. The high-level statechart model invokes lower-level modules capturing the molecular simulation of the pathway involved in chemotaxis, as well as solvers tackling the metabolic modelling. The invocations are carried out based on the statechart's active state configuration. The total combined model is fully executable. We have implemented the model using Rhapsody (Harel & Gery 1997) for the statecharts, StochSim (Novère & Shimizu 2001) for the molecular simulations, and Microsoft Solver Foundation (Optima; MSF08 2008) for metabolism solvers. Here we emphasize the general ideas and principles of our approach. A detailed definition of Biocharts and its semantics is beyond the scope of this paper. Building a generic tool for biological modelling that supports the Biocharts approach remains a topic for future work. A website for the Biocharts project, containing movies that show bacterial population dynamics, is available online (Kugler *et al.* 2009).

Although the chemotaxis model is presented here for illustrative purposes only, helping to explain the Biocharts approach, to the best of our knowledge, it is unique in its ability to integrate different aspects of bacterial behaviour, which were previously modelled in isolation or in a more qualitative and abstract manner, into a coherent quantitative systems-level model. Our model is derived from experimental biological data, and it uses as submodels state-of-the-art models for chemotaxis (using StochSim; Morton-Firth *et al.* 1999; Novère & Shimizu 2001) and metabolism (using flux balance analysis (FBA); Feist *et al.* 2007) that were constructed based on the experimental data. Obviously, the system-level behaviour depends on the precise way these submodels are connected, but we leave for future work the goal of demonstrating that such emerging global models are quantitatively accurate and can make new predictions that can be validated experimentally. We feel that the Biocharts approach is an important step towards achieving such an ambitious goal.

## 2. Results

### 2.1. Biological background

Bacterial chemotaxis is one of the most well-known biological subsystems, and thus serves as a potentially promising target for computational modelling, which in general requires some mechanistic understanding of the system to construct useful models.

In general, movement is thought to be a selective advantage in heterogeneous environments, where, for example, attractants (such as nutrients) and repellents (toxins) are not spread out evenly. To guide the movement in a beneficial direction, the organism must be able to sense changes and gradients in its environment. Most bacteria are generally considered to be so small that it is usually not possible for them to sense gradients across their diameter. Thus, they are forced to sense temporal changes in concentrations during their motion.

Movement in bacteria is usually composed of a repeated sequence, consisting of a relatively straight motion followed by a reorientation to another direction. By making the reorientations more frequent when the gradient is in an undesirable direction and less frequent when it is in a beneficial direction, the bacterium can relocate itself to a more suitable place.

Our model focuses on *Escherichia coli*, which typically has five to eight flagella (helical semi-rigid filaments), each driven by its own motor complex. Viewed from behind the cell, a motor can rotate clockwise (CW) or counterclockwise (CCW), resulting in the corkscrew form of the filament. If all the motors (and flagella) rotate CCW, they form a bundle that propels the cell forward. If one or more of the flagella change direction of rotation, the bundle breaks and the cell tumbles, causing a change in its current direction of movement, after which all the motors return to CCW rotation and push it forward in the new direction. The chemosensory system of *E. coli* affects the motion by increasing or decreasing the tumbling frequency, based on the changes in extracellular chemoattractant concentrations.

**Figure 1.**
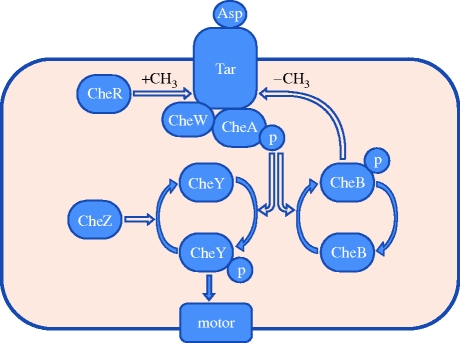
Simplified chemotaxis pathway. The shaded area represents intracellular space.

The direction of rotation of the motors is controlled by the network of chemotactic proteins. A simplified picture of the chemotactic network is shown in figure 1. Coarsely, the functioning is such that an extracellular ligand aspartate (Asp) binding the Tar complex decreases the autophosphorylation (P) of protein CheA, reducing the amount of phosphorylated CheY (CheY-P), which makes the motors less bound by this protein, and has the effect of increased CCW rotation, which causes longer runs of the bacterium. In addition, the phosphorylation of CheB is reduced, which indirectly (but in concert with CheR) has the effect of increasing the methylation level of the receptor Tar complex. This, in turn, causes increased CheA autophosphorylation. This feedback mechanism allows for adaptation to different levels of a chemoattractant. Ligand dissociation from the receptor, resulting from a decrease in the concentration of the chemoattractant, has roughly the opposite effect, causing a CW rotation, hence a tumbling behaviour. For additional details, see Wadhams & Armitage (2004).

### 2.2. Modelling

#### 2.2.1. Chemotaxis

Modelling bacterial chemotaxis has been studied widely for many years (see Tindall *et al.* 2008*a*,*b*). Both the structural and biochemical steps in the pathway have been characterized in detail. *In silico* models built based on this knowledge have been observed to capture the basic chemotactic behaviour faithfully. Bacterial chemotaxis has been used to study robustness in Barkai & Leibler (1997) and Alon *et al.* (1999) and as a test case for computational biology modelling tools, e.g. AgentCell (Emonet *et al.* 2005). Thus, it makes sense to select such a model to form part of the lower-level basis of a two-tier Biochart model and to use the combined result to study new system-level interactions.

**Figure 2.**
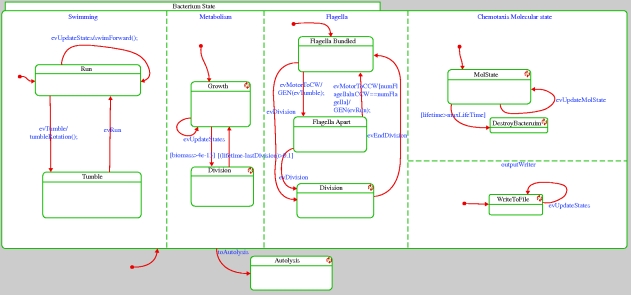
The bacterium statechart.

The model we have built describes basic chemotactic behaviour of bacteria, combined with their metabolic response. The overall system-level behaviour is modelled using the object-oriented version of the statecharts language (Harel 1987; Harel & Gery 1997), and is implemented in Rhapsody (Harel & Kugler 2004). Each relevant biological subsystem (environment, bacterium, motor, etc.) is represented by a class. The multiplicities of objects (such as the number of bacteria and the number of motors a bacterium has) are easy to control by creating or deleting (statically or during runtime) instances of these classes. The dynamic behaviour of instances of a class is prescribed by states whenever this makes sense and is represented by the statecharts. Examples include the swimming state of a bacterium (Run versus Tumble; figure 2) and the direction of rotation of a single motor (CW versus CCW).

**Figure 3.**
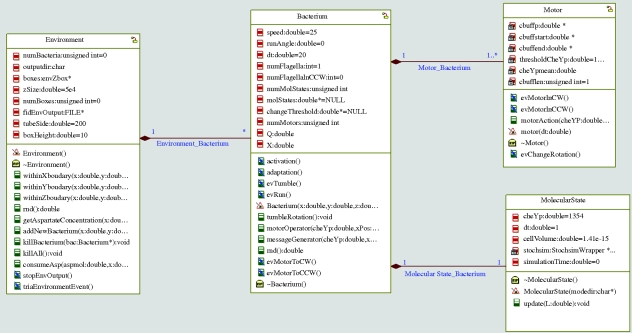
Structure of the bacterium model. Each box represents a class and the lines between the boxes depict their associations. For example, the many-to-one line between bacterium and motor represents the fact that a bacterium has several motors, which are part of it, and that each motor is part of exactly one bacterium. The attributes and methods of classes are listed inside the boxes.

The main component of our system is the Bacterium class, which contains Motor and MolecularState classes (figure 3). The Motor class represents the flagellar motor that drives the movement of the bacteria. Our model allows the instantiation of several such motors, which is required, since, for example, *E. coli* can have up to eight motors (Levin *et al.* 1998). Motor switching is controlled by CheYp concentration and for this we used a two-threshold, hysteresis model (Morton-Firth & Bray 1998). The thresholds were selected to produce, in uniform concentration environment, rotation characteristics (average tumbling and run times, CW bias) matching, as closely as possible, those measured experimentally. As reported in Weis & Koshland (1990), despite differences in the tumbling frequency, the chemotactic response towards attractants is still about the same. Thus, because our model of the chemotactic machinery is supposedly accurate, even without tuning the motor parameters to perfect concordance between the measured and simulated tumbling frequencies, the chemotactic response should be very close to real bacterial behaviour.

The molecular-level pathway behaviour is modelled in the class MolecularState, and it activates the StochSim (Novère & Shimizu 2001) simulation engine. The differential equations representing the interactions of the network and the initial levels of molecules are handed to StochSim, which simulates the state of the network using a stochastic simulation method (Novère & Shimizu 2001) that is similar to the Gillespie algorithm but capable of handling multi-state molecules (e.g. ones with multiple methylation states such as the Tar receptor here) much more efficiently and has been shown to perform well in chemotactic simulations (Morton-Firth & Bray 1998). Relevant parameters are sent to StochSim and the results of the calculations are obtained on the fly. Our intracellular simulations are non-spatial; i.e. they assume fast enough diffusion of substances within a cell. Simulating the spatial aspects within a cell would be a nice possible extension of our model.

The detailed chemotaxis modelling in our work follows Morton-Firth *et al.* (1999). In real cells, properties such as steady-state behaviour and adaptation time can vary as the protein concentrations are not equal from cell to cell but the precision of adaptation is robust (Alon *et al.* 1999), suggesting that for a realistic model an important aspect is adaptation, and otherwise we can allow variations in behaviour. The model we use allows for practically perfect adaptation, and thus arguably captures the most crucial characteristics of a chemotactic network.

The Tar complex is assumed to have two conformational states, active and inactive (Asakura & Honda 1984; Barkai & Leibler 1997). For ligand–receptor interaction (i.e. aspartate–Tar complex), we assume equilibrium binding, so that the fraction of bound receptors in inactive conformation is *p* = [*L*]/(*K*_D_ + [*L*]), where [*L*] denotes the ligand concentration and *K*_D_ = 1.71 µM denotes the dissociation constant. Similarly, for receptor occupancy p* in active conformation, we use the dissociation constant *K*_D_* = 12 µM (see Emonet *et al.* 2005).

#### 2.2.2. Metabolism

Our model enables one to investigate the influence of metabolic activity (growth, ATP requirements for swimming, etc.) on chemotactic behaviour. For metabolism, we used a whole-cell stoichiometric model (Feist *et al.* 2007). Such models do not incorporate kinetic information and they are usually simulated using FBA (Varma & Palsson 1994; Bonarius *et al.* 1997; Edwards *et al.* 2001), which assumes the metabolic network to be in a steady state. Formally, FBA involves the following.

— A steady-state assumption d**c**/d*t* = **Sv** = **0**, where **c** is a vector of metabolite concentrations, **S** is the stoichiometric matrix and **v** is a vector of reaction rates. This assumption states that each metabolite is consumed at the exact same rate as it is being produced, thus there is no accumulation of any metabolite.
— Boundaries for reaction rates *α* ≤ **v** ≤ *β*. Setting lower bounds allows us to define some reactions as irreversible, and upper bounds can be defined based on, for example, information about enzyme kinetics. These bounds are also used to define the inputs to the network; i.e. the medium composition of the environment.
— A definition of the objective function as a linear combination of reaction rates, i.e. **f · v**. Most often this is the biomass function, which is determined from experimental data to reflect the energy and biomass constituent needs of the cell. This has been found to be a reasonable assumption, particularly for *E. coli* under certain growth conditions (Edwards *et al.* 2001; Schuetz *et al.* 2007).

Thus, the FBA is a linear programming problem and we solve it using Microsoft Solver Foundation (MSF08 2008). To account for the fact that the extracellular concentrations can change, the metabolic steady state was updated (using the normal FBA) in response to such changes. This is called *dynamic flux balance analysis* (dFBA) in Mahadevan *et al.* (2002). The condition we use for updating was that a certain amount of time had elapsed since the last update or that the input concentration changes had exceeded a given threshold. At each iteration, the aspartate consumption was calculated as *Δ*[Asp] ≈ *v*_Asp_ex__ *X*(*t*)*Δt* and biomass formation as *X*(*t* + *Δt*) = *X*(*t*) + *μ*(*t*)*X*(*t*)*Δt*, where *v*_Asp_ex__ is the rate of aspartate uptake, *Δt* is the time between iterations, *X*(*t*) is the amount of biomass at time *t* and **μ**(*t*) is the biomass growth rate.

Glucose uptake rate was modelled as in Mahadevan *et al.* (2002), with the reaction rate being *V*_Glc_max__[Glc_ex_]/(*K*_m_ + [Glc_ex_]) (mmol/g_DW_h), where the maximum value is *V*_Glc_max__ = 10 mmol/g_DW_h, *K*_m_ = 0.015 mM (experimental values) and [Glc_ex_] is the extracellular glucose concentration (Mahadevan *et al.* 2002; Wong *et al.* 1997). Aspartate uptake was modelled in a similar manner using experimental values obtained from Schellenberg & Furlong (1977).

We also modelled the division of bacteria by considering a biomass threshold for cell division. If a bacterium exceeds this threshold it enters a cell division state and divides its biomass into two by creating a new instance of bacterium.

We chose to use a stoichiometric metabolic model using FBA instead of trying to model with, for example, ordinary differential equations (ODEs), because it allows us to model a more complete metabolism and to simulate most of the metabolic functions and behaviours of a cell. In addition, the dFBA approach is well suited to our purposes since it works in a state based manner, where each steady state is a state of its own. It is also a relatively fast operation to perform, in comparison with large ODE models. Moreover, FBA in its objective function inherently contains an implicit turning-off of most of the unused reactions. This could also be done with an ODE model but would appear to be considerably more difficult and require many more parameters. Our objective function is also very easy to modify during the simulation, to correspond to, for example, different growth phases.

FBA methods have been observed to produce results close to experimental ones (Edwards *et al.* 2001) and the model we use is comprehensive and well tested, so that the results are bound to be fairly accurate. The inevitable errors in our metabolism modelling are likely not to have a large effect on the phenomena we wish to model here: chemotaxis driven by changing chemoattractant concentrations resulting from metabolism consuming these attractants. In our current study, the most crucial factor is the fact that the aspartate is consumed from the environment, with the rate being a function of its concentration. Since this rate is experimentally determined, it should be sufficiently correct.

#### 2.2.3. Environment

The environment in our model consists of a tube modelled as having a rectangular cross section. Using a tube as the environment was motivated by real chemotactic assays, such as those described in Adler (1966). For simulating spatially varying concentrations, the tube was divided into slices along the longitudinal axis. The walls of the container were considered to be reflective boundaries for the bacteria. Initially, the tube was set to have a uniform minimal media with aspartate added at a concentration of 1 µM. Owing to the metabolic activity of the bacteria, the aspartate concentrations within each sub-box can vary. Diffusion was modelled using Fick's law.

In our model, the environment subsystem serves as one of the links through which the chemotaxis and metabolism subsystems communicate. Specifically, the metabolism changes the local concentrations within the environment that have an effect on the chemotaxis. On the other hand, chemotaxis moves the bacterium in the environment to areas with possibly different concentrations, which affects metabolism.

### 2.3. Utilizing Biocharts

There are several features of the Biocharts method that make it especially useful for the type of biological modelling described here.

The object-oriented nature of the Biocharts language allows specifying behaviours on a class level and then creating multiple objects of this class that interact to produce the emerging biological behaviour. In our chemotaxis model, this is used on several levels, by having several motors for each bacteria and modelling bacterial populations by instantiating multiple bacteria, which can later go on and divide.

The framework allows for easy crosstalk between subsystems. Different subsystems can, for example, generate events that are handled by the responsible subsystems, and subsystems can query each other through an interface. In our case, for example, the molecular-level simulation is responsible for simulating the level of CheYp, which is then sent to the motor controllers (state Flagella in figure 2), which in turn trigger an event in the corresponding motor if the level thresholds are crossed. This event is then received by, for example, the subsystem responsible for the physical movement of the bacterium (state Swimming in figure 2).

**Figure 4.**
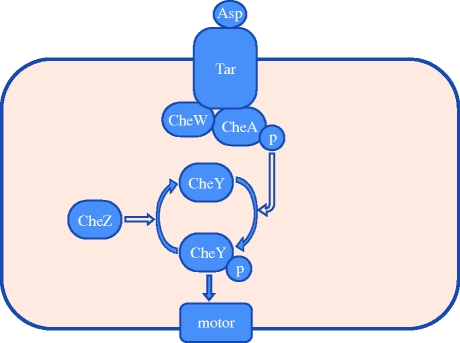
Simplified chemotaxis pathway for the *R*^−^ *B*^−^ strain.

Mutations can have a significant effect on chemotaxis. A natural way to capture this in our model is to add to the bacterium statechart orthogonal states for each of the potential relevant mutations. The StochSim code would then be activated with a modified set of reactions, where, for example, a reaction may be removed if the protein altered by the mutation originally participated in the reaction but does not participate in the mutated strain. The effect of a mutation can also be modelled by a change in the reaction rate. Two interesting mutants in our model are the *R*^−^ *B*^−^ and the *T*^−^ *W*^−^ *Z*^−^ strains (Levin *et al.* 1998). The first of these lacks the methylating enzyme CheR and the demethylating enzyme CheB, while the second lacks the Tar receptor, CheW and CheZ. A diagrammatic representation of the pathway for the *R*^−^ *B*^−^ mutant appears in figure 4.

**Figure 5.**
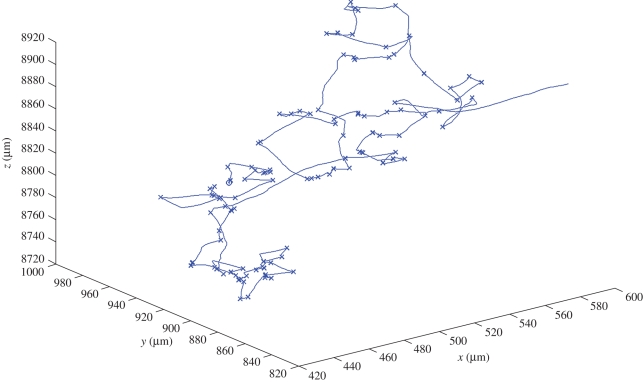
Graphical depiction of the execution of the model for a single wild-type bacterium. The starting point is marked with an ‘o’, and ‘x’s mark tumbles. Coordinates are with respect to the walls of the tube. Simulation time was 100 s.

**Figure 6.**
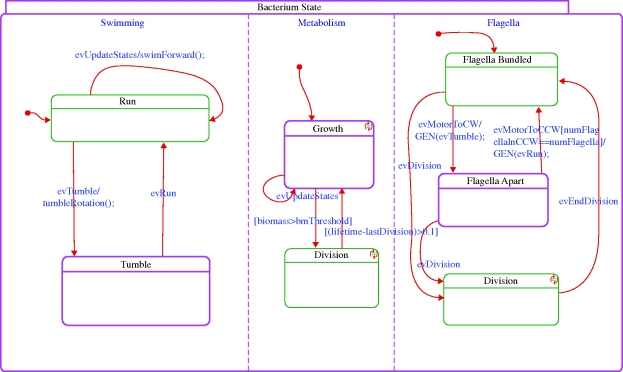
Snapshot of the statechart of a bacterium at runtime. Each of the states can contain code or a lower level model implementing the biological behaviour. For example, the Metabolism state activates the FBA model solved using Microsoft Solver Foundation. The highlighted boxes correspond to the states in which the model is at this particular time. For example, here the bacterium is tumbling and growing and its flagella are apart.

**Figure 7.**
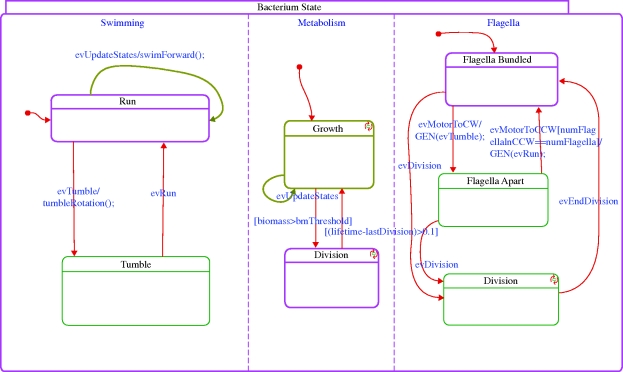
Incorporating metabolism and division. The state of the bacterium during a division phase (indicated by the highlighted Division state that was entered after the biomass passed a critical value and the bacterium starts dividing). Here the bacterium is in the Run state, is dividing and its flagella are bundled.

At runtime, we can observe the model's behaviour and can investigate the state of the different components. A graphical depiction of a sample execution of a single wild-type bacterium appears in figure 5. The path of the bacterium according to the simulation in three-dimensional space is shown; positions in which the bacterium is in the tumble state and reorients the movement direction are marked by x. Snapshots of the statechart of a bacterium during the execution are shown in figures 6 and 7. Using the orthogonal state feature of the statecharts language (i.e. ‘and’ state), figure 6 shows a bacterium simultaneously in the Tumble state of the Swimming component, in the Growth state of the Metabolism component and in the Flagella Apart state of the Flagella component. The Metabolism state is responsible for running the FBA method, which solves and computes, among other variables, the biomass. The growth of the biomass above a given threshold triggers a transition to state division, which causes the bacterium to divide, as shown in figure 7.

**Figure 8.**
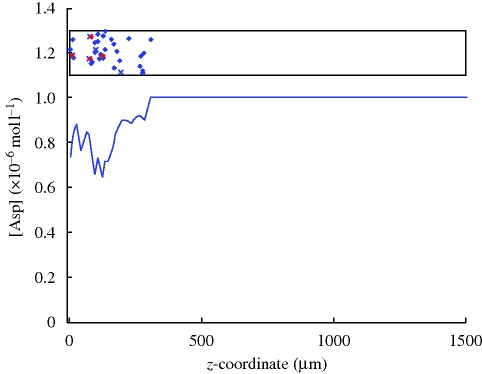
Depiction of the environmental aspartate concentration during an execution of the model on a population of bacteria in a tube. The box in the top portion of the figure represents the tube; the diamonds are bacteria in their running state and the ‘x’s are bacteria in their tumble state. The diameter of the tube is 200 µm. Bacteria shown in red were born as the result of divisions taking place within the last 1.5 s. Initially the size of the population is 30 bacteria. Aspartate concentration inside the tube is shown by the curve beneath the box. The figure represents the state of the system after 40 s of simulated time.

The Metabolism state is also responsible for interacting with the environment, by affecting the local concentrations of different substances based on the rates of consumption of the metabolites. This is illustrated in figure 8, where the model has been executed for a population of bacteria in a rectangular tube, as described above. The motivation for such simulations is that in chemotactic assays bacteria often form progressing bands, which result from the bacteria consuming a chemoattractant in their environment, thus creating a gradient along which the population starts to move (Adler 1966).

Our model allows one to study this phenomenon both on the population level and on the single bacterium level, using the ability of Biocharts to support capturing the mutual effects of different pathways and biological processes. In our case, we want to integrate the effects of metabolism on the actual bacterial chemotaxis pathway and their effect on population-level dynamics. Movies showing bacterial population dynamics are available online (Kugler *et al.* 2009) and illustrate some of the current capabilities of the Biocharts model. Among other things, our approach allows for experimenting with simulations using several different parameter values and replacing certain modules by more abstract representations, which can give valuable insight into the functioning of the full system.

As an example, we can follow in detail the process of band formation and monitor properties such as spatial and temporal distribution of tumbling bacteria or CheYp concentrations within each bacterium. For example, a study of the online simulation movie suggests that at the beginning of the simulation, the fact that there are many bacteria consuming aspartate from a common small area, the effect is a rather rapid depletion of aspartate, which results in a higher proportion of the bacteria being in their tumbling state. This has the effect of these bacteria staying in the same area while consuming aspartate, so that they eventually create a large concentration gradient.

It is important to recall that the main aim of this paper is to introduce the modelling language, and hence the scope of our modelling effort is focused on the movement of a single bacterium or of small populations (up to 100 bacteria). Creating simulations for larger populations of bacteria (Tindall *et al.* 2008*a*) can be supported as a natural extension, as was done in some of the previous statechart modelling efforts carried out in the last-listed author's research group (Efroni *et al.* 2007; Setty *et al.* 2008). However, the additional pathway-related computations that the Biocharts require add to the challenge of running such massive simulations efficiently. Ideas for future work on handling this complexity include approximating the behaviour described in particular states by simpler and less computationally intensive behaviour, caching results of computations and distributing the model to run on a cluster.

## 3. Discussion

The Biocharts language and its underlying approach were designed to tackle some of the fundamental challenges in making the modelling of complex biological systems more accessible and mainstream.

In general, as the biological system becomes more complex, suggested models and diagrams grow in size and can become extremely difficult to understand. In an attempt to help alleviate this problem and ease both the modelling and the comprehension of the models, we suggest a general solution in which the model can be decomposed naturally on a behavioural basis (not necessarily a structural one). To that end, we use a language genuinely intended for reactive behaviour, which supports a variety of temporal relationships between ‘pieces’ of low-level behaviour, including sequentiality, concurrency, inclusion and more. Wise use of the hierarchy of states and orthogonal components available in statecharts allows one to construct more succinct representations of the model in a natural way. We are thus using constructs and concepts that have proven to be powerful and natural in software and systems engineering. The combination becomes a state-based description of the reactive dynamics of a complex biological system, with the molecular dynamics—possibly continuous and feedback-oriented in nature—taking place on the lower tier of our two-tier approach, that is, within any of the states at any level of the statechart hierarchy.

In some of the existing notations, the same network may in principle be activated in several different ways, but it is far more difficult to specify if all possible combinations can actually occur in reality or under which conditions. The essence of the Biocharts approach is to enable a flexible, modular and hierarchical breakup of networks and pathways based on their behaviour, in any way that is deemed natural by the experts of the subject matter. Thus, by embedding the relevant parts of the networks and their behaviour within the states, the upper-tier statecharts can be made to capture the dynamic and temporal inter-relationships between those network portions. Moreover, this intra-state network information can be incorporated as it becomes available and for any set of experimental conditions.

If the behaviours described within the states are given in some diagrammatic form, say of the Kitano or Kohn flavours, Biocharts become doubly visual, but this need not be the case. Our proposal is for a language consisting, first and foremost, of statecharts, with some form of bioprocesses embedded within. These could be given, for example, as process algebras or calculi (Regev *et al.* 2001; Ciocchetta & Hillston 2008), as Petri nets (Reddy *et al.* 1996) or directly as differential equations. Anything that provides dynamics for molecular interactions or pathways is acceptable as the ‘internal language’ of the proposed combination. The new combined two-tier language inherits the power of statecharts to talk about modularity of the dynamics, time, causality, concurrency, and in general to provide answers to ‘what happens when’ questions. It may be beneficial to use the Biocharts approach with a scenario-based language, e.g. live sequence charts (Damm & Harel 2001), as the high-level modelling language (Kam *et al.* 2008), which remains a future research direction.

A crucial advantage of embedding the network and pathway pieces in the statecharts is that as long as the former are endowed with enough dynamic information, the entire model of the biological system is fully executable, as we illustrated here. This allows one to use the existing theory and tools for the simulation, analysis, verification and visualization of complex biology. We hope that it will also encourage the development of more powerful such tools, tailored specifically for biological modelling.
